# Thoracoscopic Surgical Treatment of Spontaneous Pneumothorax: Selection of Surgical Therapy According to Thoracoscopic Findings

**DOI:** 10.1155/DTE.1.165

**Published:** 1995

**Authors:** Y. Sugamura, M. Jibiki, H. Ikari, T. Nakamura, H. Kawabe, T. Ishibashi, T. Kunizaki

**Affiliations:** Department of Surgery Sasebo Chuo Hospital 4–5, Tonoo Cho Nagasaki Sasebo 857 Japan

## Abstract

We report our experience with thoracoscopy used for the treatment of spontaneous pneumothorax and idiopathic emphysematous bullae. Fifty-one patients with pneumothorax were admitted to the hospital and received a pleurography and CT scanning before thoracoscopy. End-GIA resection or end-loop ligation were used alone or in combination, with or without laser coagulation. Only one patient developed recurrent pneumothorax, whereas another required repeated resection. Our results indicate that surgical treatment of pneumothorax using thoracoscopy results in a rapid expansion of the lung, minimum postoperative pain, early hospital discharge, and return of normal activity.


*Diagnostic and Therapeutic Endoscopy*, 1995, Vol. 1, pp. 165–168
Reprints available directly from the publisher
Photocopying permitted by license only

© 1995 Harwood Academic Publishers GmbH
Printed in Malaysia

### Thoracoscopic Surgical Treatment of Spontaneous Pneumothorax: Selection of Surgical Therapy According to Thoracoscopic Findings

Y. SUGAMURA, M. JIBIKI, H. IKARI, T. NAKAMURA, H. KAWABE, T. ISHIBASHI, and T. KUNIZAKI

*Department of Surgery, Sasebo Chuo Hospital, 4–5, Tonoo Cho, Sasebo City, Nagasaki, Japan 857*

*(Received February 21, 1994; in final form, August 15, 1994)*

We report our experience with thoracoscopy used for the treatment of spontaneous pneumothorax and idiopathic emphysematous bullae. Fifty-one patients with pneumothorax were admitted to the hospital and received a pleurography and CT scanning before thoracoscopy. End-GIA resection or end-loop ligation were used alone or in combination, with or without laser coagulation. Only one patient developed recurrent pneumothorax, whereas another required repeated resection. Our results indicate that surgical treatment of pneumothorax using thoracoscopy results in a rapid expansion of the lung, minimum postoperative pain, early hospital discharge, and return of normal activity.

**KEY WORDS:** Thoracoscopy, pneumothorax, emphysematous bullae, thoracotomy, cardiothoracic surgery

### INTRODUCTION

Endoscopic surgery is changing the history of surgery. Tremendous changes have recently occurred in the field of thoracic surgery as a result of advances in fiberoptic technology, video imaging, and electronic instrumentation. Video-assisted thoracic surgery or thoracoscopy has gained a prominent position with widespread applications reaching far beyond the pleural space for which it was once developed.

We have used thoracoscopic procedures for treatment of patients with spontaneous pneumothorax as early as September 1991. We report here our experience and policy regarding indications, techniques and results using several thoracoscopic procedures.

### PATIENTS AND METHODS

Fifty-one patients with spontaneous pneumothorax were treated in our department using different thoracoscopic procedures since September 1991. It was our policy to perform thoracoscopy (Olympus Thoracoscope A-5254, Japan) as a first-choice procedure without a chest tube drainage. In most patients, thoracoscopy was performed shortly after admission. In the presence of severe symptoms, pleural exsufflation or drainage was first initiated, followed by thoracoscopy within 24 hours. On the other hand, the patient was kept under medical observation without surgical intervention in the presence of mild symptoms, occurrence of spontaneous pneumothorax for the first time, the bleb was not evident and lung collapse was less than 10%.

Management of spontaneous pneumothorax involved several steps. The first step necessitated a proper selection of patients. In contrast to other nonoperative methods used for treating spontaneous pneumothorax, there are relatively few absolute contraindications to thoracoscopic surgery. This is because thoracoscopic procedure could be easily converted to an open thoracotomy once the initial thoracoscopic evaluation of the lesion and surrounding tissues is completed. Patients with poor general health were considered unsuitable, because the procedure was performed under general anesthesia.

The second step in the management of these patients involved radiologic examination of the chest. When the presence of bleb(s) could not be confirmed with a plain chest x-ray, two additional examinations using pleurography (Fig. 1) and CT scanning (Fig. 2) were performed before thoracoscopy. Excellent pleurography was ob-

Address for correspondence: Yoji Sugamura, MD, Department of Surgery, Sasebo Chuo Hospital, 4–5, Tonoo Cho, Sasebo City, Nagasaki, Japan, 857.

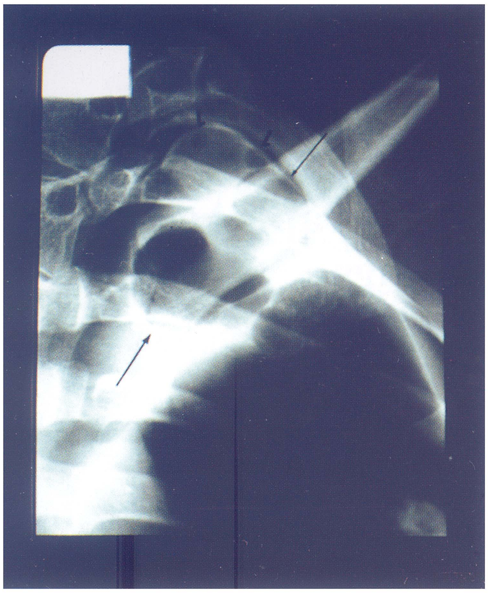

**Figure 1** Pleurography clarifies blebs at the apex of left lung and air bubbles in the pleural cavity.

tained by turning the patient into various body positions. 0.5 to 1.0 mL/kg body weight of non-ionic contrast medium Iopamidol (Iopamiron 150) was injected into the pleural space through a fine elastic needle or chest tube.

Once the type of lesion was determined radiologically, an appropriate surgical procedure was selected based on thoracoscopic findings. Fig. 3 illustrates the different methods used for treatment in our patients. At first, we considered that the endo-loop ligation was a useful treatment for an isolated bleb 3.0 cm or less in diameter (Type I). However, a further improvement in surgical techniques and instruments resulted in expansion of indications of endo-GIA to include Type I cases also. Small diffuse blebs of Type III were coagulated using the nontouch method with low YAG laser pulses.

General anesthesia was performed with a univentilation tube intubation in order to collapse the lung and allow adequate field of vision. Surgery was performed with the patient in the lateral position. The standard point of entry was on the anterior axillary line in the fourth intercostal space. The lung surface and parietal pleura were initially viewed with the thoracoscope connected to a camera and external videomonitor. In this regard, no blind spots in the thoracic cavity were detected with the recent use of a flexible thoracoscope (LTF, Olympus, Japan). Two trocars were inserted to introduce different instruments. This was usually performed in the fourth or fifth intercostal space and on the posterior axillary line or midclavicular line, depending on the location of the bleb. Correct positioning of these cannulas was vital for a proper thoracoscopy. A quick control of bullae was accomplished with an endoloop using chromic catgut or PDS (size 0, 18", 45cm, Ethicon Inc., U.S.). Loops, together with their pushrod, were loaded into the suture applicator and then into the cannula. Once the loop was in the pleural cavity, the bleb was grasped within the loop and held steady while the one-way slipknot was tightened. The endo-GIA endoscopic linear cutter (Ethicon Inc., U.S.) featured a long staple line, one-handed operation, and parallel jaw closure. Bigger or complicated bullae were resected using endo-GIA, whereas small and diffuse bullae were coagulated with YAG laser pulses. A 12-mm trocar was required to insert the endo-GIA. Air leakage was checked by applying a small amount of water into the pleural space. Finally, an 18 Fr intercostal drainage tube was inserted through the anterior cannula site, and the skin approximated with

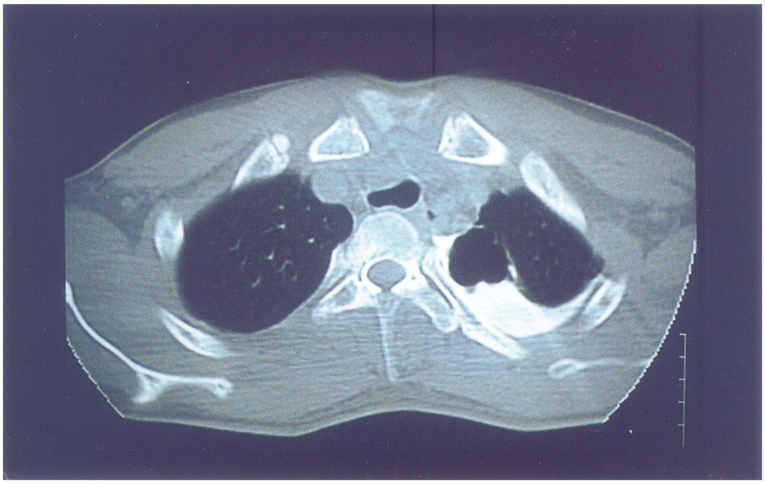

**Figure 2** A computerized tomography (CT) of the chest after pleurography showing blebs in the contrast medium.

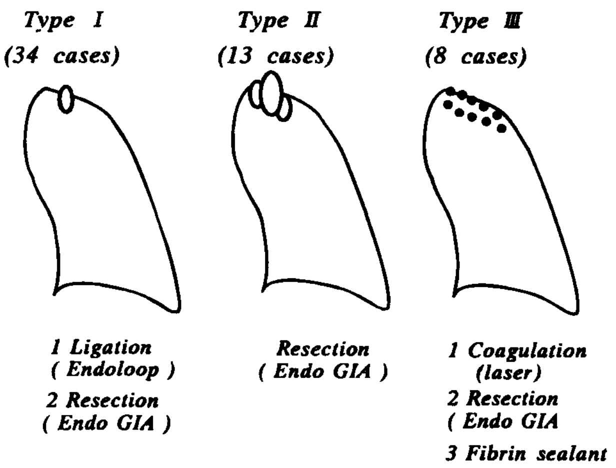

**Figure 3** Methods of surgical approaches and number of cases, according to thoracoscopic findings.

**Table 1** Characteristics of Patients

| | |
|---|---|
| Age | 14–72 yrs (average, 29 yrs) |
| Sex | Males, 45 |
| | Females, 6 |
| Primary spontaneous pneumothorax | 28 |
| Recurrent spontaneous pneumothorax | 23 |
| Site of lesion | Right 20 |
| | Left 31 |
| Operative time | 27–175 min (average 62 min) |
| Days of hospital admission | 3–16 days (average 8 d) |

**Table 2** Endoscopic operative methods.

| | |
|---|---|
| Resection (endo-GIA) only | 25 |
| Ligation (endo-loop) only | 9 |
| Resection and ligation | 12 |
| Resection and laser coagulation | 3 |
| Ligation and laser coagulation | 1 |
| Resection, ligation and laser coagulation | 1 |

interrupted sutures. A drainage tube was placed for the first 24 hours.

### RESULTS

Fifty-one patients with spontaneous pneumothorax were treated between 1991 and 1994 with thoracoscopic procedures described above. They included 45 men and 6 women patients between 14 and 72 years of age (mean, 29 years). The pneumothorax was on the left side in 31 patients. It was the first episode in 28 patients, whereas 23 patients had a recurrent pneumothorax (Table 1). Twenty-five patients were treated by endo-GIA only, whereas 9 patients were treated by end-loop ligation. Laser coagulation therapy was used in three cases as a precautionary measure (Table 2). We applied fibrin glue to the pulmonary surface of Type III pneumothorax on several occasions to protect against recurrence. Conversion to an open thoracotomy was required in two patients. This was due to a difficulty in separating hilar vessels in one case, whereas second patient had complicated bullae formation with multiple tissue involvement.

### RECURRENCE AND COMPLICATIONS

During a follow-up ranging from 1 to 29 months (median, 11.4 months), only one patient developed recurrence 1 month after thoracoscopic ligation. Thoracotomy at that time revealed rupture of a new small bulla, which was successfully coagulated with low power YAG-laser pulses. No major operative or postoperative complications were experienced except in one patient. The patient had thoracoscopic blebectomy using a single endo-GIA. Hemosputum and air leakage continued for 6 days after the operation. Bronchoscopy at that time revealed bleeding from the upper segment of the left lung. Insertion of the thoracoscope through the original site revealed a small amount of blood clot present on the edge of the GIA suture site. The cause of hemosputum and prolonged air leakage was considered to be GIA stapler malfunction, and another endo-GIA was performed. The patient returned to work 9 days after the second operation.

### DISCUSSION

Spontaneous pneumothorax tends to recidivate when treated conservatively. Only recently have some authors published their experiences using thoracoscopy for pneumothorax.[1–3] These reports conclude that this technique is helpful as an alternative therapeutic approach for pneumothorax. Whereas some authors perform only thoracoscopy at the first relapse of pneumothorax, others, including ourselves, prefer to perform thoracoscopy when the condition is diagnosed for the first time.[4] In experienced hands, thoracoscopy should be a safe method of endoscopy without serious complications. In our opinion, thoracoscopy represents a simple procedure and as invasive as the insertion of a pleural drainage tube.

Criticism may be raised regarding justification of pleurography for all patients. When the lesion is not clear, especially in the presence of a collapsed lung, aspiration of the free air becomes necessary to obtain a clear outline of the visceral pleural surface. This is usually performed using a fine elastic needle or a chest tube before the application of the contrast medium for pleurography. In our

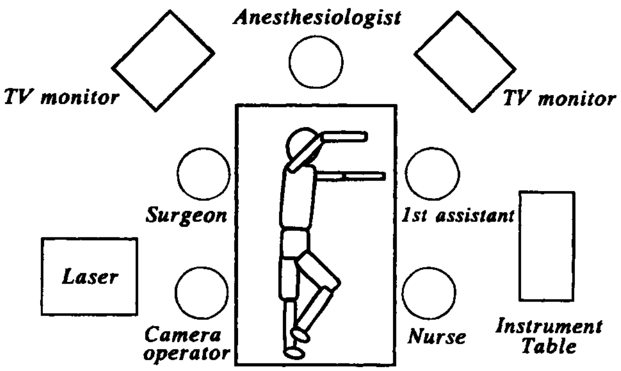

**Figure 4** Operating room layout.

experience, pleurographic findings corresponded well with the inspection of pleural surface and the lung during thoracoscopy. Occasionally, it also may be possible to detect air bubbles during pleurography, thus pointing to the site of air leakage.

Thoracoscopic excision of blebs as described in the present report offers an ideal approach to this common disorder. Performed thoracoscopically, excision of all blebs using a stapler offers the most definitive treatment of the pathology. Thoracoscopy seems to offer advantages similar to those of laparoscopy. With the development of endoscopic stapling devices, it appears that thoracic surgeons can perform operations in a visceral manner equivalent to traditional open procedures.

Several authors reported the usefulness of fibrin sealant in persistent, therapy-resistant, complicated pneumothorax.[5] The same method was used in one patient presenting with recurrence in the present study. The fibrin glue was applied to the cut edge of endo-GIA. It should be cautioned that pleurodesis may increase the morbidity associated with thoracoscopic surgery and reduce its effectiveness.[6] It is our policy not to establish any pleurodesis by abrasion of the parietal pleura or application of chemical materials. We also believe that it is preferable to preserve the natural anatomy during such procedures. Any future thoracic condition requiring surgery will be more easily accessed when the pleural space is preserved.

Our postoperative assessment included gathering information through questionnaires. Almost 95% of these patients answered that they felt hardly any postoperative pain or that it was less than they had anticipated. Most patients returned to work within 2 weeks after the operation. In general, all patients were satisfied with the endoscopic procedure and would recommend such surgery to their relatives or friends (Table 3).

**Table 3** Questionnaire

| | |
|---|---|
| I) | How was your postoperative pain? |
| II) | When could you return to work? |
| III) | Did you play any sport after operation? |
| IV) | Do you mind your operation scar? |
| V) | Are you satisfied with the endoscopic surgery you had? |
| VI | Would you recommend this endoscopic surgery to your relatives or friends? |

We conclude that thoracoscopic surgical treatment of spontaneous pneumothorax produces favorable results especially in adolescent patients with isolated bullae.

### CONCLUSIONS

1. Thoracoscopic surgical treatment was performed in 51 patients with spontaneous pneumothorax.
2. The surgical technique should be based on thoracoscopic findings.
3. Only a single case with recurrence and another with a minor postoperative complication were reported.
4. The advantages of thoracoscopic surgical treatment included a rapid full expansion of the lung, a small skin scar, minimum postoperative pain, early hospital discharge and return to normal activity.

### REFERENCES

1. Vershoof AC. Thoracoscopic pleurodesis in the management of spontaneous pneumothorax. Respiration 1988;53:197–200.
2. Nathanson LK. Videothoracoscopic ligation of bulla and pleurectomy for spontaneous pneumothorax. Ann Thorac Surg 1991;53:316–19
3. Wakabayashi A. Thoracoscopic ablation of blebs in the treatment of recurrent or persistent spontaneous pneumothorax. Ann Thorac Surg 1989;48:651–53
4. Vanderschueren RGJRA. The role of thoracoscopy in the evaluation and management of pneumothorax. Lung 1990;168(Suppl.):1122–25
5. Hansen MK. Spontaneous pneumothorax and fibrin glue sealant during thoracoscopy. Eur J Cardiothorac Surg 1989;3:512–14
6. Hauck H. Complicated pneumothorax: Short- and long-term results of endoscopic fibrin pleurodesis. World J Surg 1991;15:146–50
7. Bagnato VJ. Thoracoscopic treatment of spontaneous pneumothorax without pleurodesis: a preliminary report. Giorn Chir 1992;13:137–39

